# Hypertrophied Tonsils

**Published:** 1885-07

**Authors:** A. W. Calhoun

**Affiliations:** Professor of Diseases of the Eye, Ear and Throat in the Atlanta Medical College, Atlanta, Ga.


### HYPERTROPHIED TONSILS.

A CLINICAL LECTURE BY A. W. CALHOUN, M. D., PROFESSOR OF DISEASES OF THE EYE, EAR AND THROAT IN THE ATLANTA MEDICAL COLLEGE, ATLANTA, GA.

Reported for THE ATLANTA MEDICAL AND SURGICAL JOURNAL.

One among the most frequent causes of chronic deafness is the long-continued irritation in the eustachian tubes from hypertrophied tonsils in children. The case I now present you is a typical case of this common trouble.

It is a disease peculiar to childhood, beginning very early in life, and is frequently congenital and hereditary. Almost always the disease attacks both tonsils—very rarely does it attack only one.

The tonsils in the healthy condition are about the size of an ordinary almond without the hull, and are situated on each side of the base of the tongue between the pillars of the fauces.

This case is a typical form of the disease, the glands being so large as almost to touch each other. In nearly all such cases, characteristic symptoms accompany the disease, viz.: difficult breathing, especially at night, sleeping with the mouth open be cause of the obstruction in the posterior nares, caused by the enlargement, muffled voice, more or less chronic pharyngitis, and as the result of breathing through the mouth, cracked and broken teeth. The alternate contact of hot and cold air upon the teeth causes the disintegration of them, but the great danger is the deafness that usually follows long-continued hypertrophied tonsils, by reason of the extension of the inflammation into and more or less pressure upon the mouths of the eustachian tubes.

Children sometimes outgrow the disease, but while they are outgrowing it the danger to the hearing is constantly increasing ; hence the necessity of active treatment in all children who have enlarged tonsils and the necessity of disregarding the advice to "let them alone, they will outgrow it."

It is just as necessary for good hearing that air should circulate unobstructedly through the eustachian tubes as it is necessary that air should pass unobstructed into the lungs for good breathing. It is too often the case that we see young adults with more or less impairment of hearing, as the result of a previously long-continued mechanical irritation in the throat and post-nasal region from enlarged tonsils, even though the enlargement at this period—the adult period—may have entirely disappeared. Proper attention to the tonsils at the proper time in childhood, would have preserved the hearing and relieved the child of a great annoyance.

The causes of this disease are numerous, but in children, when not congenital or hereditary, it is the result of frequent catarrhal sore throat. The prognosis of all these cases is favorable if the proper treatment is instituted in time, but if not, the child frequently falls into general bad health with cough, catarrh, deafness, etc., and the appearance not unusually reminds one of approaching consumption.

### TREATMENT.

The treatment is very simple, but very radical. Only one thing is of any service, and that is excision of the tonsils. In my hands all other treatment has been not only of temporary benefit, but a great trouble and annoyance both to the child and the parents.

I read a pamphlet by a Frenchman some years ago, who advanced the original idea that removal of the tonsils "unmanned the patient, and that all the powers of procreation were lost." He failed to say what effect it would have upon the females. I mention this statement as a mere curiosity, for there is not the slightest evidence to corroborate the assertion. One of the mos fruitful patients I have ever had was a young man whose tonsils I removed just previous to his marriage. Several children had gladdened his home when I last heard from him.

The operation of excision is simple. It is made with a pair of curved Vulsellum forceps and a curved probe-pointed bistoury with a long handle. I have long since discarded all tonsilotomes—they are invariably a little too large or a little too small to suit the tonsils. The best plan is to sit the patient on the lap of an assistant with the face towards the light and have the head held steady from behind. The operator should always sit in front. If the child does not object, it is always well to have the assistant cover the eyes so as to shut out from view the movements of the operator, sight of the instruments, etc. Have the child to open its mouth wide, depress the tongue with a tongue depressor, or with the finger, grasp the tonsil firmly with the forceps and gently pull it towards the median line of the throat, pass the bistoury underneath the tonsil and cut from below upwards, so that the intended line of incision will not be hidden by the flowing blood, and regulating the amount of tonsil to be removed by the size of them. The intention should be only to remove so much of the tonsil that when recovered it will be about its normal size. During the operation the child's struggles will be great, but quick manipulation will enable the operator to finish before the child fairly discovers what is going on. Now comes the "tug of war," if it is desirable to remove the other tonsil; the child knows what has happened and what must happen on the other side, and resists any further procedure by every imaginable means. If the parents have not sufficient influence to induce the child to open its mouth, my rule is to give them a few inhalations of chloroform, just sufficient to obtund the sensibility of the child, then force the mouth open and seize the tonsil and proceed as with the first one. The hemorrhage that follows the operation is not great, usually stopping of its own accord in five minutes, but if it should not, it is easily checked by means of gargling salt water, or, if necessary, a few drops of muriate tinc. of iron in water may be gargled. Out of several hundred operations I have seen but one case of serious hemorrhage and that was secondary.

The after treatment consists of salt water gargle, fluid diet and good care for three or four days; the parts soon heal. It is well for every operator to become ambi-dexter, as by that means he can save a great deal of trouble.

The good effects of the operation are seen almost immediately. The child talks, breathes and sleeps better than for months past, and often the impaired hearing begins at once to improve, though ordinarily the improvement in hearing is a matter of several months time.

Don't hesitate, gentlemen, to remove tonsils when they are very much enlarged and are a mechanical obstruction, and don't have any fears of "unmanning" your patient.

